# Prescribing Behavior Change: Opportunities and Challenges for Clinicians to Embrace Digital and Mobile Health

**DOI:** 10.2196/17281

**Published:** 2020-08-04

**Authors:** Anish Agarwal, Mitesh Patel

**Affiliations:** 1 Department of Emergency Medicine University of Pennsylvania Philadelphia, PA United States; 2 Perelman School of Medicine University of Pennsylvania Philadelphia, PA United States; 3 Leonard Davis Institute for Health Economics University of Pennsylvania Philadelphia, PA United States; 4 Center for Health Care Innovation Penn Medicine Philadelphia, PA United States; 5 Nudge Unit Penn Medicine Center for Health Care Innovation University of Pennsylvania Philadelphia, PA United States; 6 Department of Medicine, Crescenz Veterans Affairs Medical Center Philadelphia, PA United States

**Keywords:** digital health, behavior change, mobile health, patient-centered data collection

## Abstract

Individual behaviors impact physical and mental health. Everyday behaviors such as physical activity, diet, sleep, and tobacco use have been associated with a range of acute and chronic medical conditions. Educating, motivating, and promoting sustained healthy behaviors can be challenging for clinical providers attempting to manage their patients’ health. The ubiquity and integration of mobile and digital health devices (eg, wearable step counters, smartphone-based apps) allow for individuals to generate and record enormous amounts of patient-generated health data. Research studies have begun to reveal how mobile and digital devices offer promise in motivating individual behavior change but they have not had consistent results. In this viewpoint, we discuss the potential synergy of digital health modalities and behavioral strategies as an approach for clinicians to prescribe, motivate, monitor, and sustain healthy behaviors. We discuss the strengths, challenges, and opportunities for the future of promoting health behaviors.

Everyday health behaviors such as diet, physical activity, tobacco use, and medication adherence contribute significantly toward long-term health [1]. While clinicians certainly recognize the importance of these behaviors, they often feel paralyzed when it comes to addressing them [2,3]. Most commonly, clinicians will attempt to change behavior through education (eg, highlighting the importance of a healthy diet and regular exercise) [4]. While it may be helpful for a patient to hear this from their clinician, it is often not enough to lead to sustained behavior change [5].

What if clinicians could more easily prescribe, motivate, and sustain behavior change, similar to how they prescribe medications and monitor changes in laboratory test results for diabetes or high cholesterol? Regular exercise is just as important for both of these conditions, yet clinicians lack an easy mechanism to offer therapy or motivation as well as monitor progress. Previously, the only way to obtain information on daily health behaviors was through self-reporting, which is often unreliable and inconsistent. Nearly 80% of US adults now carry a smartphone [6] and the ubiquity of these digital technologies as well as wearable devices allows for seamless, passive, and remote tracking of behaviors such as physical activity or sleep patterns [7]. Emerging literature has begun to describe the applications of digital health modalities across varying specialties and patient populations [8,9]. The strength of digital health may lie in its ability to offer clinicians a means to individualize, engage, and support an individual’s behavior change continuously as compared with intermittent office visits or activity logs. Moreover, these strategies may be used worldwide, including in low- and middle-income countries where mobile phone ownership is high and access to care may be limited. Effective implementation of these approaches, combined with strategies rooted in behavioral science, could improve health across populations. However, they remain relatively unexplored and underutilized by clinicians.

Several recent studies reveal promising opportunities. In a randomized trial of patients with ischemic heart disease, those provided with a wearable device for 6 months had no meaningful change in physical activity [10]. However, patients with personalized goals coupled with loss-framed financial incentives accumulated more than 100 miles of increased steps with sustained activity levels during the following months, when incentives were stopped. In another study, families from the Framingham Cohort were randomly assigned to use an activity tracker, select a step goal, and receive daily feedback or do each of those in addition to being enrolled in a game that incorporated behavioral insights (eg, precommitment, loss aversion, and peer support) into the design [11]. Families that played the game achieved activity goals at significantly higher rates during the 12-week intervention with sustained differences relative to control during the 12 weeks after the game ended. These trials reveal an important insight. Simply providing an activity tracker did not lead to meaningful changes in activity. However, combining the technology with a behavioral strategy led to significant changes that were sustained in the postintervention period. Although longer-term studies are needed, these trials demonstrate the potential use of digital health to effectively motivate populations toward long-term healthy behaviors such as physical activity, dietary changes, or smoking cessation. These studies offer early insights on an approach that, when integrated into practical clinical workflows, offers patients an additional method of capturing and managing their own data. In addition, this approach would enable clinicians to deploy evidence-based behavior change strategies.

How can clinicians incorporate these types of approaches within their practice? First, clinicians must be able to interpret and manipulate data collected on patient behaviors within the electronic health record (EHR). Granular data reflecting daily step counts are likely to become overwhelming. Instead, longitudinal trends and inflection points may reveal opportunities for intervention and longer-term monitoring. Some institutions enable patients to send their information to their EHR through Apple’s Healthkit [12], but these data need to be presented in more actionable ways and integration models are needed to reflect the growing number of devices produced by different companies (eg, FitBit, Samsung, Google). Clinicians need the ability to automate and individualize tailored behavior change techniques to patient needs in real time. Behavior change will likely require an approach which evolves with the patient over time to maintain long term results. Digital interfaces have a unique capacity for personalization due to patient-level sociodemographic factors. Additionally, accessing and understanding these data and the methods of integrating behavior change into practice must begin during medical training. Medical trainees, now adept with digital technologies, are primed to learn how to best weave in behavior change strategies when managing individual or population health.

Second, clinicians and patients must be able to trust the data collected to inform health decisions [13]. Many consumer-grade health technologies have not been extensively evaluated for large-scale use, and their accuracy, validity, and reliability remain unknown. Some types of data may require a higher level of validity. For example, algorithms that not only monitor heart rate but also attempt to identify abnormal heart rhythms [14] may require larger clinical trials than studies testing the accuracy of tracking steps. Data security is also important, particularly for more sensitive patient information.

Third, once the infrastructure is in place to collect, display, and interpret data, then clinicians need a mechanism and reimbursement structure for prescribing and monitoring behavior change. This will depend on sufficient evidence to identify effective approaches and EHRs to allow for ordering of behavioral interventions to be just as simple as ordering a medication. Reimbursement mechanisms will need to be in place to support these initiatives. There are examples of insurance coverage for effective behavioral interventions such as cardiac rehabilitation and the diabetes prevention program [15,16]. Yet, even these interventions are often not offered to all patients who could benefit from them. The additional time and effort clinicians use for digital health monitoring and the accompanying decision making will need to be accounted for and will vary. Similar to “interactive” automobile or life insurance policies, which use smartphone data to offer incentives for safe driving practices or physical activity, health insurers could stand to benefit from accessing enrollees’ data from behavior change programs.

Finally, to achieve success in prescribing behavior change, clinicians will need to use effective, evidence-based methodologies to motivate and sustain behaviors. Prescribing behavior change through digital health methods will need real-time, seamless, and continuous quality improvement to evaluate which mechanisms are working (or failing) to produce meaningful outcomes. As the capabilities of technology evolve and the approaches to monitoring health data grow, research will need to prioritize efforts in investigating the sustained effectiveness of varying behavioral approaches [17].

Motivating lifestyle-related changes in patients remains a critical yet challenging task for clinicians. Not all behavior change strategies are created equal and studies have demonstrated heterogenous results in the ability to maintain long-term results. Coupling these strategies with digital health may provide a means to build a multimodal approach to sustained outcomes. However, there are some obstacles to utilizing digital health to monitor, motivate, and support these behavior changes. Despite the seeming ubiquity of technology, not all individuals have access to smartphones or wearable devices. Many digital health companies have created partnerships with health insurers to expand access and provide a means to monitor chronic disease and drive healthy habits. Furthermore, the technologic landscape is rapidly changing and will require clinicians to be nimble in their understanding of the use and limitations of digital health technologies. Data validity and integration will need to be continuously studied, upheld, and monitored to ensure safe and effective use. Qualitative research from key stakeholders highlights the need for good data management and quality assessment [18]. The digitization of medicine will offer clinicians an additional mode of delivering care and monitoring health. The role of other stakeholders (eg, insurers or employers) will also be important to study. As patient-generated digital data continues to evolve, health systems must focus their efforts on embedding these digital modalities into clinical practice alongside behavioral approaches. Medical training should incorporate these approaches and empower clinicians with an additional toolkit to motivate and monitor behavior change in their patients.

The predominant approach to promoting lifestyle modification has relied on educating patients. The exponential growth, capacity, and functionality of digital health may help provide a pathway toward prescribing behavior change just like a clinician would prescribe a statin. While several challenges exist, technology will continue to evolve rapidly. Developing behavioral strategies which are evidence-based and practically scalable will help clinicians focus on implementation in their local environments. The critical next step is to better integrate and test behavioral strategies to support clinicians’ efforts to improve their patients’ long-term health.

## Abbreviations

EHR: electronic health record
